# Supplementary material for: Dissecting the Spatially Restricted Effects of Microenvironment-Mediated Resistance on Targeted Therapy Responses
Source: Cancers (Basel). 2024 Jun 29;16(13):2405. doi: 10.3390/cancers16132405 (PMC11240540; doi:10.3390/cancers16132405)
Supplement: Supplementary file 1 [file cancers-16-02405-s001.zip › cancers-3027578-supplementary.pdf]

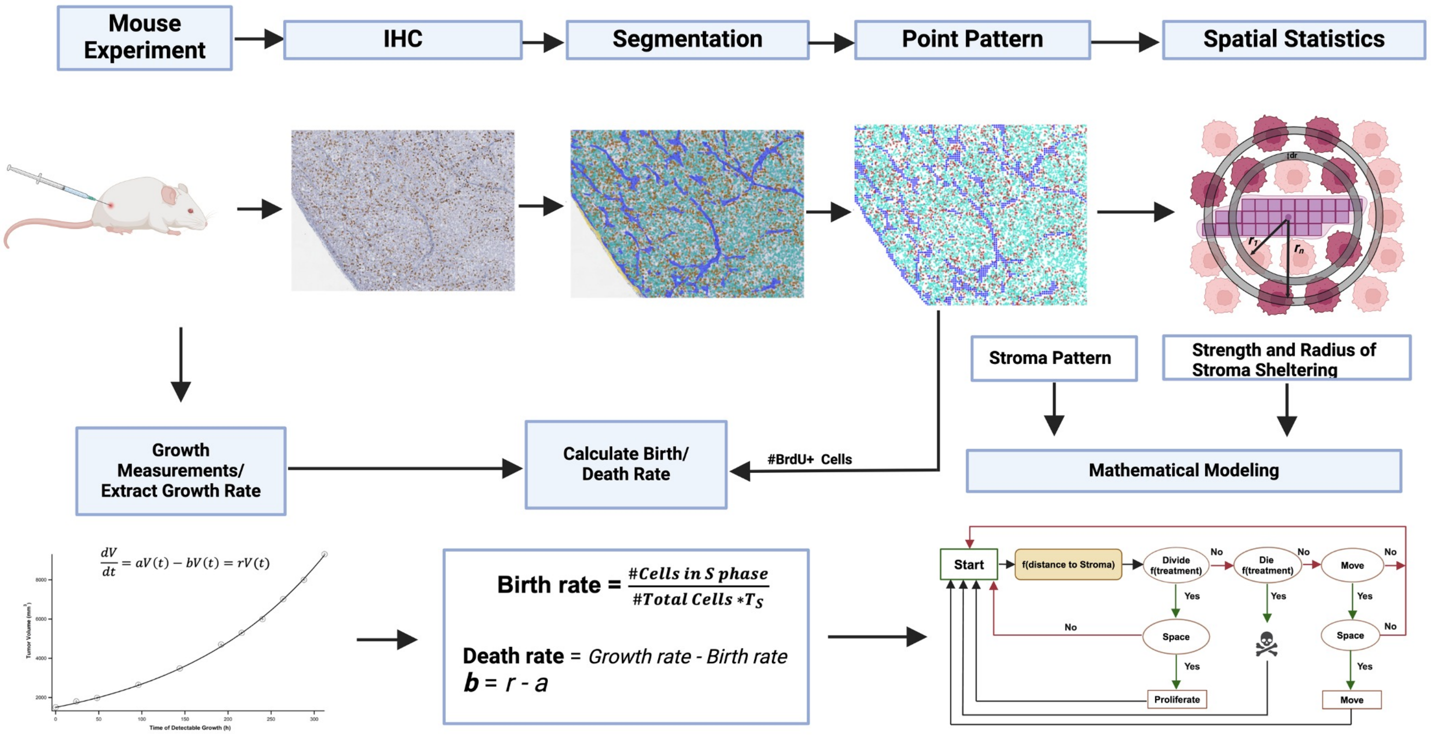

Figure S1. Detailed workflow of the approach

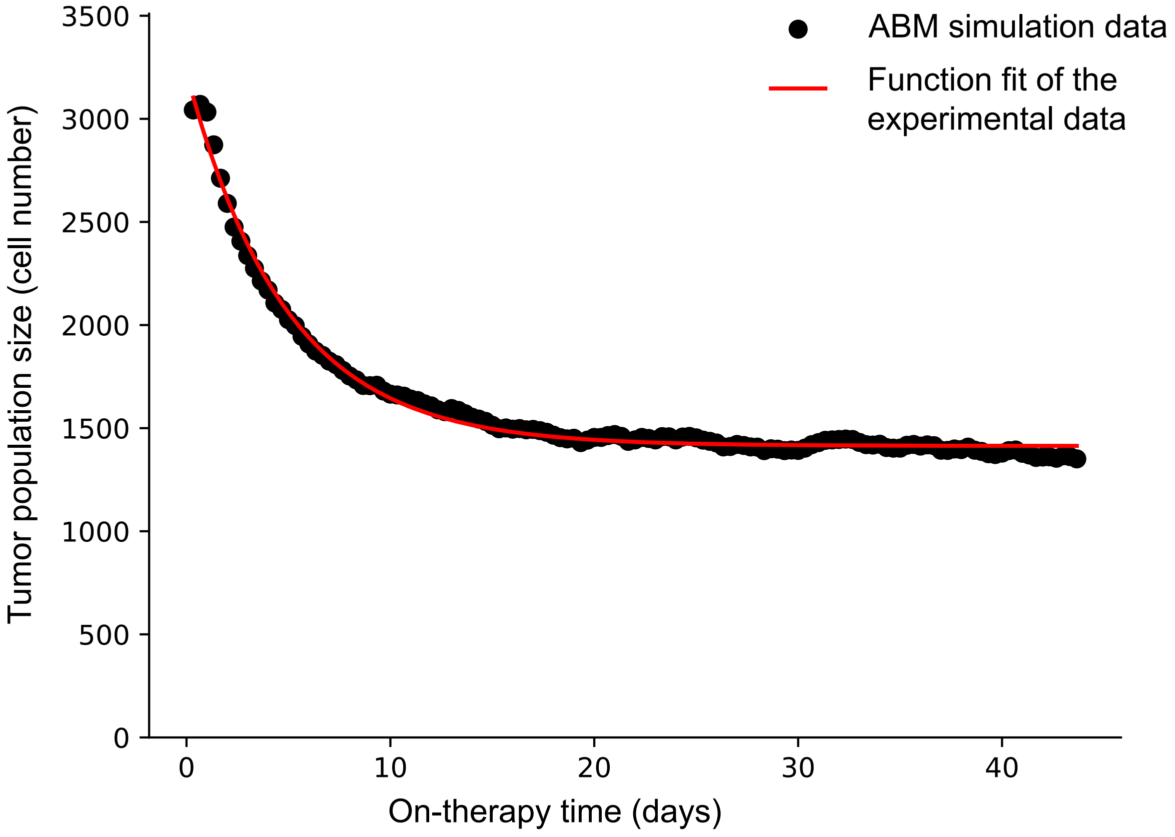

Figure S2. Comparison of the decay rate function inferred from the experimental data and the outcomes of the ABM simulations for the quadrant I. In silico data shows average of 100 simulations for quadrant I.

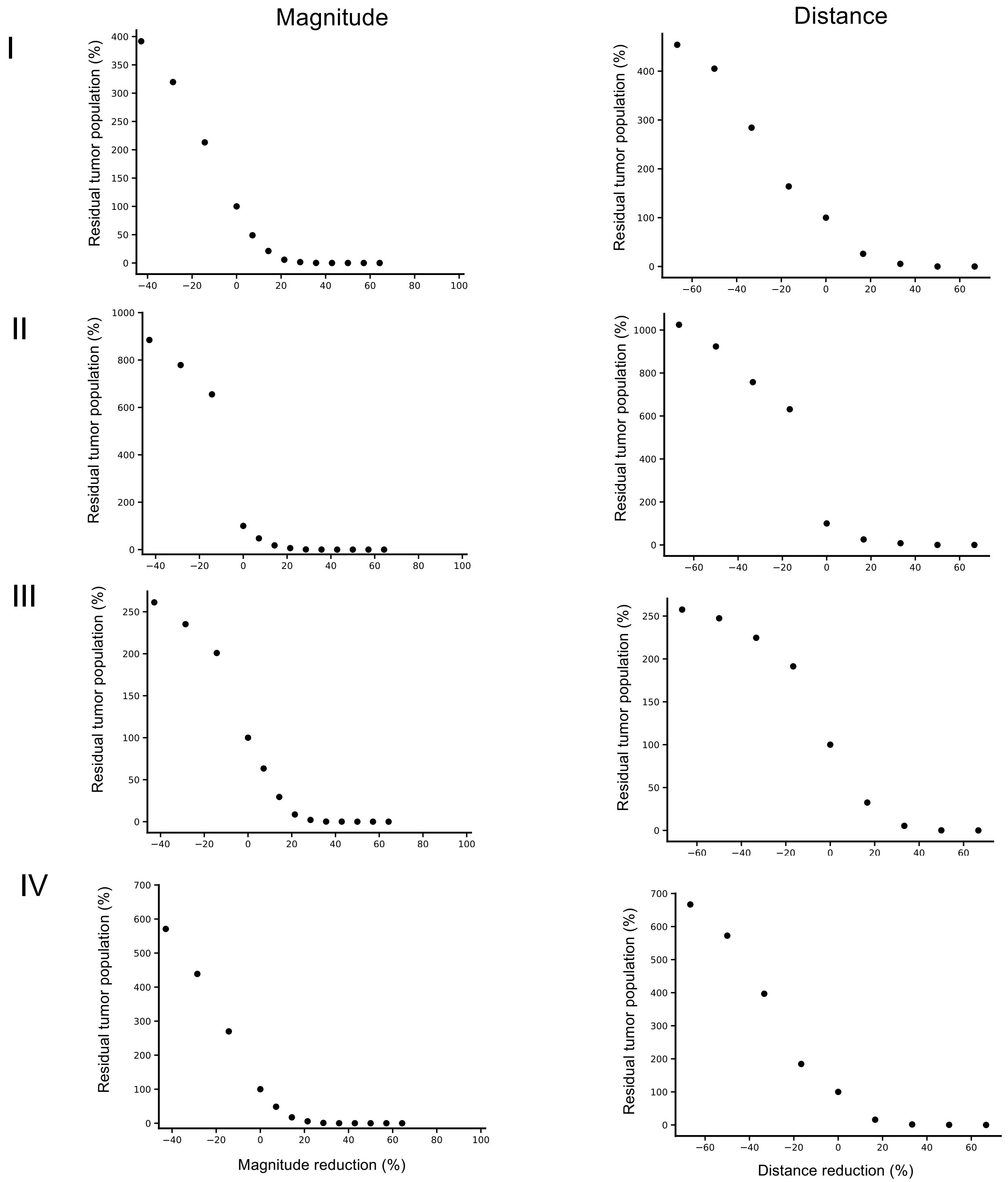

Figure S3. The impact of modulation of magnitude and distance of EMDR effects for quadrants I-IV. The data are normalized to the quadrant-specific on-treatment baselines from the experimental inferences. Individual dots represent the averages of 100 simulations for the indicated quadrants. Negative x axis values represent an increase in the parameter value.

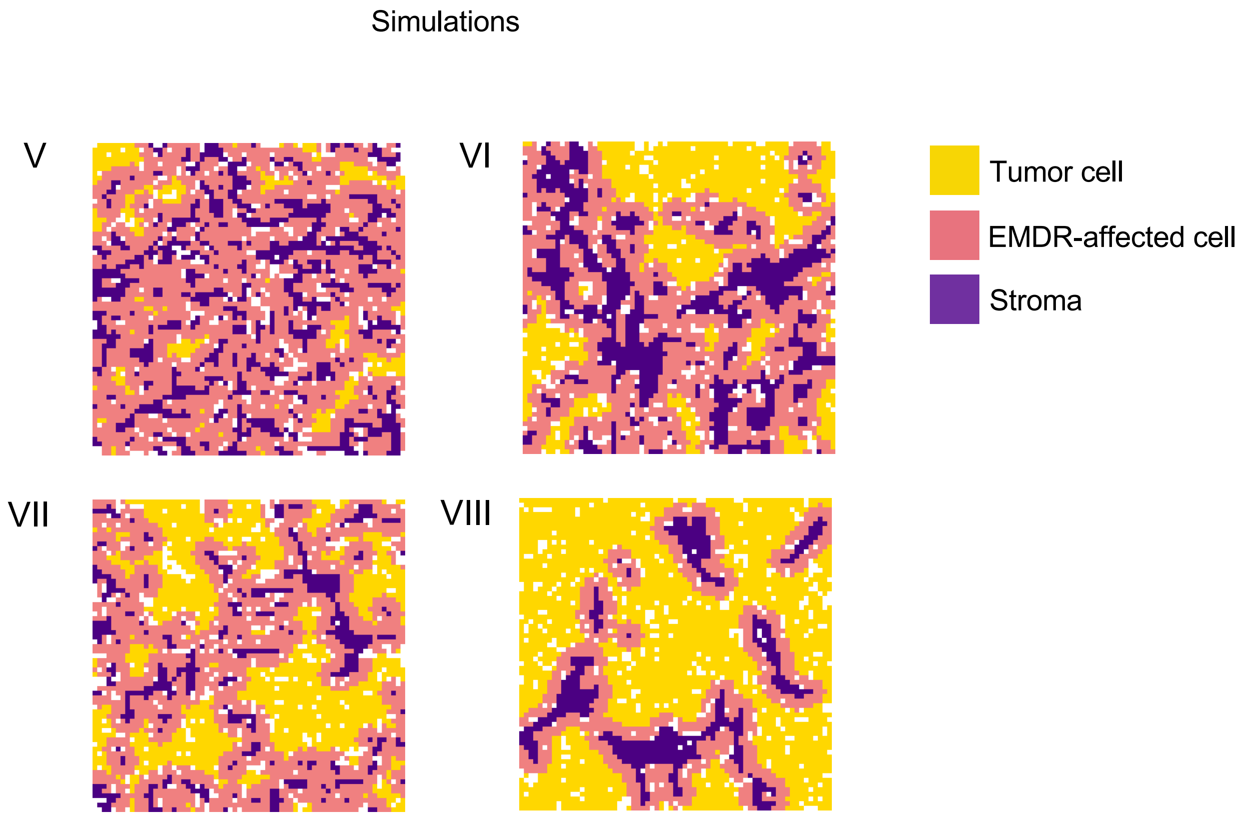

Figure S4. Representative snapshots of ABM simulations initialized with quadrants V-VIII

Tissue quadrant	I	II	III	IV
**Stroma abundance**	16%	16%	18%	35%
**Stroma dispersal**	81%	61%	88%	64%

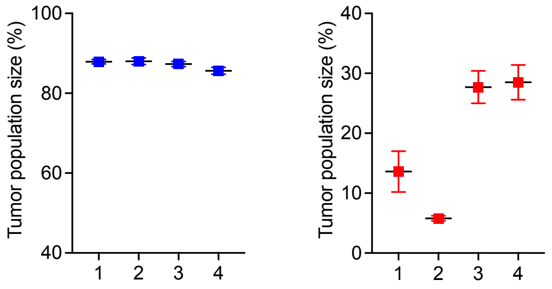

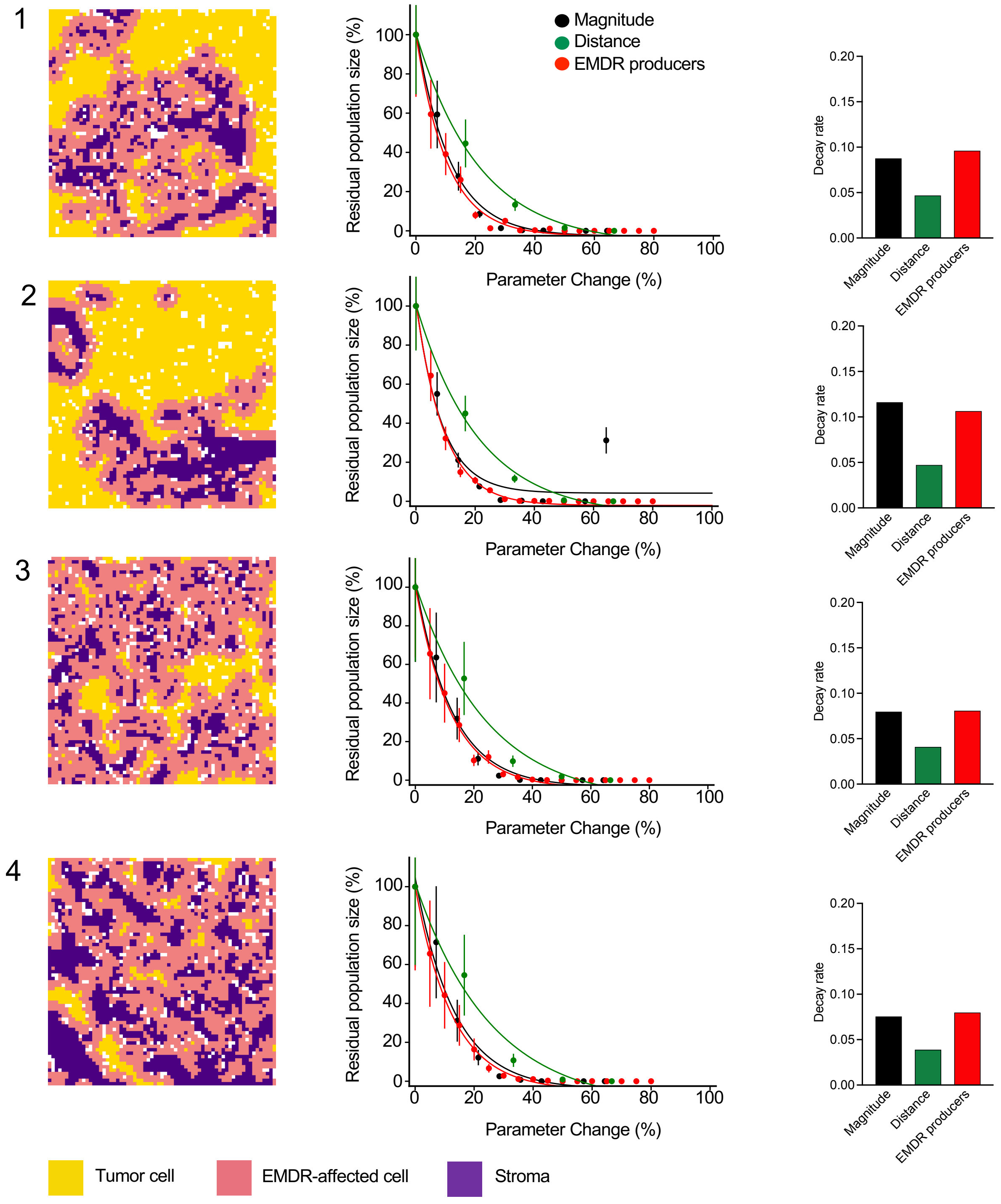

Figure S5. ABM analyses initiated with histologies from human clinical samples. The data is presented analogously to the main Figure 5.
